# Determinants of Cesarean Section among Primiparas: A Comparison of Classification Methods

**Published:** 2018-12

**Authors:** Saman MAROUFIZADEH, Payam AMINI, Mostafa HOSSEINI, Amir ALMASI-HASHIANI, Maryam MOHAMMADI, Behnaz NAVID, Reza OMANI-SAMANI

**Affiliations:** 1. Department of Epidemiology and Reproductive Health, Reproductive Epidemiology Research Center, Royan Institute for Reproductive Biomedicine, ACECR, Tehran, Iran; 2. Department of Epidemiology and Biostatistics, School of Public Health, Tehran University of Medical Sciences, Tehran, Iran

**Keywords:** Cesarean section, Primiparas, Artificial neural network, Random forest, Logistic regression, Classification

## Abstract

**Background:**

Over the last few decades, Cesarean section (CS) rates have increased significantly worldwide particularly in Iran. Classification methods including logistic regression (LR), random forest (RF) and artificial neural network (ANN) were used to identify factors related to CS among primipars.

**Methods:**

This cross-sectional study included 2120 primipars who gave singleton birth in Tehran, Iran between 6 and 21 July 2015. To identify factor associated with CS, the classification methods were compared in terms of sensitivity, specificity, and accuracy.

**Results:**

The CS rate was 72.1%. Mother’s age, SES, BMI, baby’s head circumference and infant weight were the most important determinant variables for CS as identified by the ANN method which had the highest accuracy (0.70). The association of RF predictions and observed values was 0.36 (kappa).

**Conclusion:**

The ANN method had the best performance that classified CS delivery compared to the RF and LR methods. The ANN method might be used as an appropriate method for such data.

## Introduction

Cesarean section (CS) is “one of the most commonly performed surgeries in obstetric practice” (1). In most of the countries, there has been a dramatic rise in the CS rate over the past few decades, and there is a wide variation in CS rates between countries. Based on the latest data from 150 countries throughout the world, the CS rate was 18.6% (2). The highest and lowest rate of CS was reported in Latin America and the Caribbean (40.5%) and Africa (7.3%). The rate of CS in other regions of the world were as follows: Northern America (32.3%), Oceania (31.1%), Europe (25%), Asia (19.2%) (2). According to WHO: “There is no justification for any region to have CS rates higher than 10%–15%” (3).

The CS has some complications for the mother and infant. Common maternal complication includes bleeding, infection, postpartum hemorrhage, wound infection, endometritis (4–9). Some consequences for the infant include breathing problems, low Apgar scores, premature birth from an incorrect gestational age and fetal injury (10, 11). In most cases, CS is not due to medical necessity, but factors other than medical indications are involved. Factors such as fear of delivery pain, previous complicated vaginal delivery, previous CS, lack of sufficient knowledge about normal vaginal delivery and lack of relief methods are determinant factors to choose CS (12, 13).

Classification methods are a set of data mining techniques used to predict group membership for new cases. A variety of classification methods have been introduced such as logistic regression (LR), random forest (RF), artificial neural network (ANN), support vector machines (SVM), decision tree (DR), k-nearest neighbor, and boosting. (14, 15). Lots of studies in medical and clinical area have applied classification methods (16–18).

Selecting the applied classification method is very important so that accurate classifications can lead to accurate predictions. Among different classification approaches, LR is well known and utilized due to its ease of use and interpretation. However, RF is preferable when a large number of covariates and factors are available and used. ANN as a non-linear, flexible, and general tool can detect complex nonlinear relationships between dependent and independent variables (19, 20).

According to the increasing rate of CS and its adverse consequences for the mother and child, it is important to diagnose and predict the CS delivery.

Therefore, we aimed to determine the rate of CS among primipars in Tehran, Iran, and identify factors related to it using different classification methods including LR, RF and ANN. Furthermore, this study investigates the performance of these classification methods for CS data.

## Materials and Methods

### Participants and study design

This cross-sectional study was conducted on 2120 primiparas referred to hospitals across Tehran Province, Iran affiliated to one of these four universities, Tehran University of Medical Sciences, Shahid Beheshti University of Medical Sciences, Iran University of Medical Sciences and Islamic Azad University, from 6–21 July 2015.

### Ethical approval

This study was approved by the Ethics Committee of Royan Institute, Tehran, Iran (Code: IR. ACECR. Royan.REC.1395.43). The purpose of the study and confidentiality of the data were explained verbally to the pregnant women by midwives and nurses before data collection. Moreover, written informed consent was obtained from all participants before completing the questionnaires.

### Questionnaires

For data collection, a checklist containing mother’s demographic information, obstetrical data and newborn’s information, was used. The checklists were filled out through direct interview with mothers and reviewing their cases in delivery room by a nurse or a trained obstetrician, which it included information such as mother’s age (years), mother’s education (academic، non-academic), father’s education (academic، non-academic), mother’s occupation (housewife, employed), socio-economic status (SES), body mass index (kg/m^2^), infant sex (male, female), infant weight (g), infant height (cm), baby’s head circumference (cm), type of pregnancy (wanted, unwanted), history of abortion (no, yes), history of stillbirth (no, yes), preeclampsia (no, yes), use of assisted reproductive technology (no, yes) and type of delivery (vaginal, CS). The criterion for preeclampsia was having a blood pressure reading of more than 140/90 millimeters of mercury (mmHg) and the presence of an excess of proteins in the urine (proteinuria). A principal component analysis was performed on checklists that pertained to home appliances and digital goods to determine the SES of each family.

### Statistical analysis

Data analysis was performed with IBM SPSS Statistics for Windows, ver. 22.0 (IBM Crop., Armonk, NY, USA) and R (R Core Team, 2017). To perform the classification methods and to validate the results, the test and training samples were composed randomly among cases. The results derived from the training sample (70% of cases) was then evaluated by utilizing the test sample (30% of cases). In this paper, LR, RF, and ANN were used for data analysis.

### Logistic regression (LR)

LR is one of the most common applied classification methods in medical data analysis when the response variable is dichotomous. The model can be written as:

$$\log\left(\frac{\pi }{1-\pi }\right)=\alpha +\sum_{i=1}^{k}\beta_{i}x_{i}$$

In this model the *x_i_*′ s are the covariates to classify the response and the *β_i_*′ s are the regression coefficients. The term, $\left(\frac{\pi }{1-\pi }\right)$ , indicates the odds ratio of classifying the response in category of CS than vaginal delivery.

### Artificial neural network (ANN)

ANN is an information processing method. This tool is based on human brain performance. Multilayer perceptron (MLP) is frequently used among several ANN approaches. The MLP is a combination of input, output, and hidden layers with nodes in each layer. The data is transformed between the layers through an activation function and using a degree of non-linearity. Input layer consists of all risk factors affecting the output layer (CS) with two nodes as the possible outcomes. To find the best performance of the network, a complicated non-linear mapping between input and output layers is found using the number of nodes determined empirically in the hidden layer (21).

### Random forest (RF)

RF is a collection of classification and regression trees. The trees are built by a replacement sampling of the main dataset. An “out-of-bag” sample consists of the rest of data and evaluates the performance of the trees. The trees create nodes using variables that assess the occurrence of CS and a random subset of covariates is chosen at the nodes. Selection of a covariate to split node into consequent nodes is determined by a covariate which causes the largest decrease in the Gini impurity criterion. In other words, low Gini (i.e. higher decrease in Gini) shows a main role of predictor to split and classify the response variable. Therefore, the mean decrease Gini is high as well as mean decrease accuracy. After an iteration history, the final nodes contain only cases assigned to the same classes. Averaging predictions made by lots of trees allows prediction for a case at random forest (19). Moreover, an out-of-bag error, as an unbiased estimate of the true prediction error, has been used to determine the best RF.

To check the adequacy of the models, indices such as sensitivity, specificity, diagnostic accuracy (DA), positive predictive value (PPV), negative predictive value (NPV), and the area under curve (AUC) were calculated using the observed data as the gold standard. To find the amount of agreement between the observed and predicted values, Kappa statistic was calculated.

## Results

Of 2120 pregnancies, 591 (27.9%) were vaginal and 1529 (72.1%) were CS. The unadjusted comparison of demographic variables between the two groups of vaginal and CS is shown in Table 1.

**Table 1:** Demographic and clinical characteristics of the pregnant women

***Variables***	***Vaginal (n=591)***	***Cesarean section (n=1529)***	***P-value***
	***Mean ± SD or n (%)***	***Mean ± SD or n (%)***	
Mother’s age (yr)	24.76 ± 4.66	28.25 ± 5.08	<0.001
Mother’s Education			<0.001
Non-Academic	433 (73.3)	816 (53.4)	
Academic	158 (26.7)	713 (46.6)	
Father’s Education			<0.001
Non-Academic	450 (76.1)	881 (57.6)	
Academic	141 (23.9)	648 (42.4)	
Mother’s Occupation			<0.001
Housewife	549 (92.9)	1245 (81.4)	
Employed	42 (7.1)	284 (18.6)	
SES	−0.70 ± 1.68	0.51 ± 2.04	<0.001
Mother’s BMI (kg/m^2^)	23.50 ± 4.26	24.49 ± 5.68	<0.001
Infant Sex			0.887
Male	307 (51.9)	789 (51.6)	
Female	284 (48.1)	740 (48.4)	
Infant Weight (g)	3186.88 ± 419.01	3194.38 ± 456.94	0.729
Infant Height (cm)	49.85 ± 2.65	49.83 ± 2.54	0.866
Baby’s head circumference (cm)	34.43 ± 1.90	34.83 ± 1.79	<0.001
Type of Pregnancy			0.381
Wanted	523 (88.5)	1373 (89.8)	
Unwanted	68 (11.5)	156 (10.2)	
History of Abortion			0.452
No	503 (85.1)	1281 (83.8)	
Yes	88 (14.9)	248 (16.2)	
History of Stillbirth			0.294
No	583 (98.6)	1516 (99.1)	
Yes	8 (1.4)	13 (0.9)	
Preeclampsia			0.008
No	572 (96.8)	1436 (93.9)	
Yes	19 (3.2)	93 (6.1)	
ART			0.014
No	554 (93.7)	1382 (90.4)	
Yes	37 (6.3)	147 (9.6)	

SD: Standard deviation, SES: Socioeconomic status, BMI: Body mass index, ART: Assisted reproductive technology

Mothers in vaginal group were significantly younger than CS (*P*<0.001). Academic education resulted as a significant factor for choosing CS so that 65.3% of non-academic mothers and 81.8% of academic mothers preferred CS delivery (*P*<0.001). Housewife mothers (81.8%) were significantly less interested in CS in comparison to employed (69.3%). The socio-economic score was higher in the cesarean group (*P*<0.001). The body mass index for mother in the vaginal group was 0.99 less than CS (*P*<0.001). Babies’ head circumference in vaginal deliveries was lower than CS (*P*<0.001). A larger percentage of vaginal deliveries had no preeclampsia as well as no ART in comparison to CS (*P*=0.008 and 0.014, respectively). However, infant sex (*P*=0.887), weight (*P*=0.729) and height (*P*=0.866) as well as type of pregnancy (*P*=0.381), history of abortion (*P*=0.452) and stillbirth (*P*=0.294) were statistically the same for the two groups.

The test and train samples were randomly allocated. The demographic variables are shown for these two sets of data in Table 2 which exposes that the two sets of data are statistically the same according to the demographic variables.

**Table 2:** Descriptive statistics of participants’ demographic variables and their comparison in two sets of test and train

***Variables***	***Test (n=613) Mean ± SD or n (%)***	***Train (n=1507) Mean ± SD or n (%)***	***P-value***
Cesarean Section			0.514
No	177 (28.9)	414 (27.5)	
Yes	436 (71.1)	1093 (72.5)	
Mother’s age (years)	27.37 ± 5.16	27.24 ± 5.23	0.609
Mother’s Education			0.323
Non-Academic	351 (57.3)	898 (59.6)	
Academic	262 (42.7)	609 (40.4)	
Father’s Education			0.756
Non-Academic	388 (63.3)	943 (62.6)	
Academic	225 (36.7)	564 (37.4)	
Mother’s Occupation			0.485
Housewife	524 (85.5)	1270 (84.3)	
Employed	89 (14.5)	237 (15.7)	
SES	0.198 ± 2.013	0.158 ± 2.02	0.675
Mother’s BMI (kg/m^2^)	24.544 ± 7.43	24.08 ± 4.20	0.069
Infant Sex			0.342
Male	306 (49.9)	718 (47.6)	
Female	307 (50.1)	789 (52.4)	
Infant Weight (g)	3201.34 ± 421.44	3188.60 ± 456.53	0.552
Infant Height (cm)	49.95 ± 2.50	49.79 ± 2.60	0.195
Baby’s head circumference (cm)	34.81 ± 2.01	35.68 ± 1.75	0.158
Type of Pregnancy			0.848
Wanted	547 (89.2)	1349 (89.5)	
Unwanted	66 (10.8)	158 (10.5)	
History of Abortion			0.525
No	511 (83.4)	1273 (84.5)	
Yes	102 (16.6)	234 (15.5)	
History of Stillbirth			0.351
No	605 (98.7)	1494 (99.1)	
Yes	8 (1.3)	13 (0.9)	
Preeclampsia			0.895
No	580 (94.6)	1428 (94.8)	
Yes	33 (5.4)	79 (5.2)	
ART			0.518
No	556 (90.7)	1380 (91.6)	
Yes	57 (9.3)	127 (8.4)	

SD: Standard deviation, SES: Socioeconomic status, BMI: Body mass index, ART: Assisted reproductive technology

After LR model section using the training dataset and determining the most important variables using stepwise method, the resulted model was evaluated through the test set (Table 3). The results from LR show that CS was significantly associated with mothers’ age, SES, BMI and baby’s head circumference. Accordingly, the odds ratio of CS for one year older mothers was 1.134 (*P*<0.001) so that older mothers were more interested in CS. Adjusted for other variables, a higher score of SES and BMI resulted in more preference of cesarean so that the odds of CS was 0.751 and 0.956, respectively. One centimeter increase in head circumference raised the likelihood CS by 15.3 percent.

**Table 3:** Logistic regression model results

***Variables***	***Adjusted OR (95% CI)***	***P-value***
Mother’s age	1.134 (1.085–1.185)	<0.001
Mother’s education	1.27 (0.934, 1.727)	0.432
SES	1.284 (1.133–1.455)	<0.001
BMI	1.029 (0.985–1.075)	0.200
History of stillbirth	0.942 (0.144–6.182)	0.074
Infant weight	1.00 (0.999, 1.001)	0.196
Baby’s head circumference	1.153 (1.013–1.311)	0.031

SES: Socioeconomic status, BMI: Body mass index, OR: Odds Ratio, CI: Confidence Interval

**Fig. 1:**
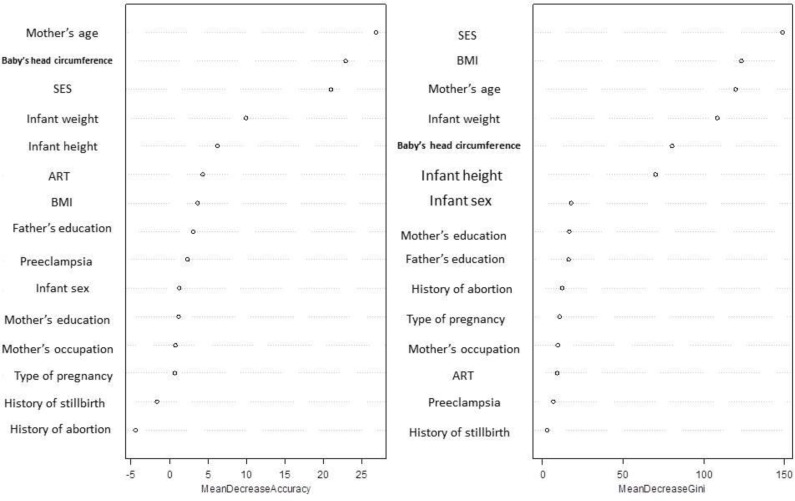
Mean decrease Gini and mean decrease accuracy of variables in random forest analysis SES: Socioeconomic status, BMI: Body mass index, ART: Assisted reproductive technology

The mean decreases Gini and mean decrease accuracy of independent variables are shown in Fig. 1. The variables mother’s age, SES, baby’s head circumference, BMI, infant weight and height were the most important predictors of CS. The out of bag error for the random forest method was 27.64%.

**Fig. 2:**
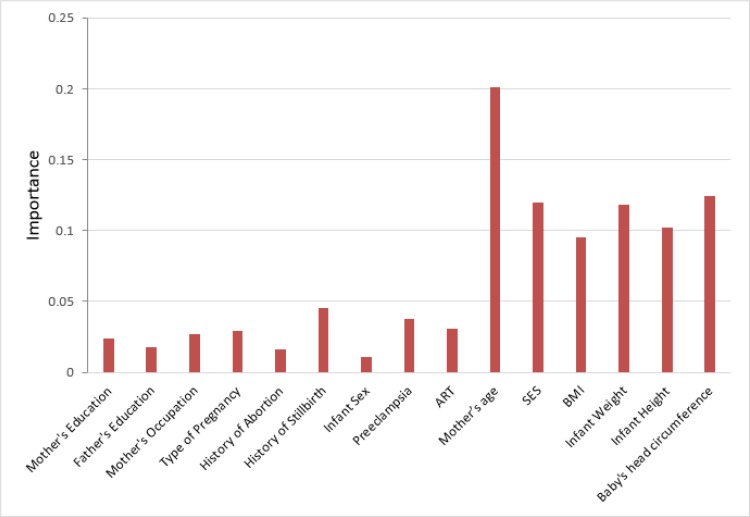
The importance of variables resulted from artificial neural network method SES: Socioeconomic status, BMI: Body mass index, ART: Assisted reproductive technology

The best ANN among several models included one hidden layer with six hidden nodes, two output, and 24 input nodes. Hyperbolic tangent and softmax were the activation functions for hidden and output layers, respectively. The importance of the variables is shown in Fig. 2 presented by scores using sensitivity analysis. The higher the variable scores, the more effective is the risk factor. Based on Fig. 2, mother’s age was the most important variable to predict CS. Moreover, the variables baby’s head circumference, SES, BMI, infant weight and height were the other important independent predictors of CS.

**Fig. 3:**
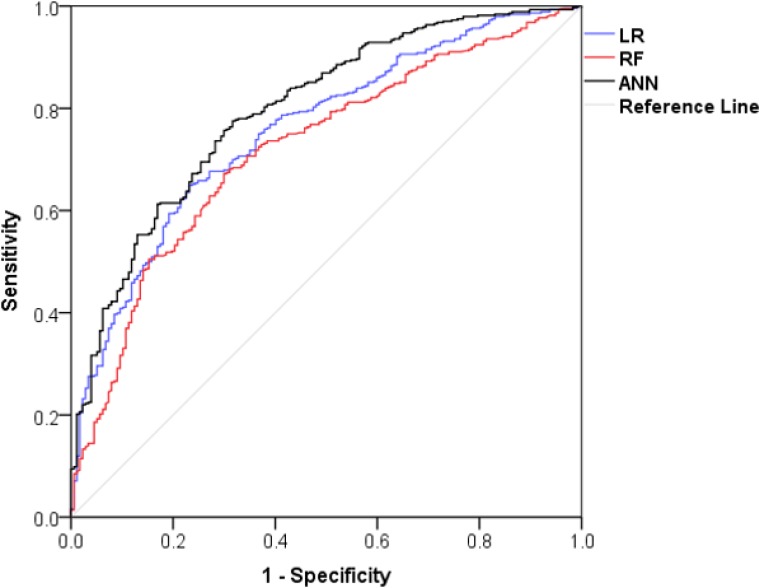
The area under curve for LR, RF and ANN methods LR: Logistic Regression, RF: Random Forest, ANN: Artificial Neural Network, AUC: Area under curve

The efficiency and accuracy of the three performed methods are compared and shown in Table 4. Tthe ANN method predicted the CS more accurate than RF and LR. All the methods had the same sensitivity while a higher specificity, PPV, NPV and accuracy for the ANN method was resulted compared to RF and LR. The agreements between the predicted and observed CS values were statistically significant. We calculated the Ø coefficient, contingency coefficient and Kendall Tau-b in order to evaluate the associations of the method’s predictions with the observed value for CS. ANN had the best performance compared to the other methods. The area under curve for the ANN method was 0.80 higher than the LR (0.75) and the RF (0.72) methods. The plot for the AUC is shown in Fig. 3.

**Table 4:** Comparison of LR, RF and ANN methods for the test sample using accuracy tools with 95% confidence interval

***Model***	***Testing Sample***		
	***LR***	***RF***	***ANN***
Sensitivity	0.67 (0.63–0.72)	0.67 (0.62–0.71)	0.67 (0.62–0.71)
Specificity	0.72 (0.65–0.79)	0.70 (0.62–0.76)	0.75 (0.68–0.81)
Positive predictive value	0.86 (0.81–0.89)	0.84 (0.80–0.88)	0.87 (0.82–0.90)
Negative predictive value	0.48 (0.41–0.53)	0.46 (0.40–0.52)	0.48 (0.42–0.54)
Accuracy	0.69 (0.65–0.72)	0.68 (0.63–0.71)	0.70 (0.65–0.74)
Kappa	0.35^*^ (0.27–0.42)	0.30^*^ (0.23–0.38)	0.36^*^ (0.28–0.43)
AUC	0.75 (0.71–0.79)	0.72 (0.67–0.76)	0.80 (0.76–0.84)
Ø coefficient	0.37^*^ (0.29–0.44)	0.32^*^ (0.25–0.40)	0.38^*^ (0.30–0.46)
Contingency coefficient	0.34^*^ (0.28–0.40)	0.31^*^ (0.24–0.37)	0.36^*^ (0.29–0.41)
Kendall tau-b	0.37^*^ (0.29–0.44)	0.32^*^ (0.24–0.40)	0.38^*^ (0.31–0.45)

LR: Logistic Regression, RF: Random Forest, ANN: Artificial Neural Network, AUC: Area under curve //

* *P*-value<0.05

## Discussion

This study aimed to determine the factors related to CS. The rate of cesarean section in Iran was higher than the rate in other countries and the one reported by world health organization (22). The rates of cesarean section were 40.5% in Latin America, 7.5% in African countries and 32.2% in the United States (2, 23). The rates in Iran during the years 2000, 2005, 2007 and 2009 were 35%, 38.4%, 45% and 47.9%, respectively (24).

Unadjusted analysis showed a significant association between CS and independent variables. However, after adjusting the effects, mother’s age, SES, BMI, baby’s head circumference and infant weight were found as the most important and affective variables on CS. In a study to investigate socioeconomic factors on CS rates, a higher SES causes higher rate of CS was found (25). This may be due to the stress and fear of delivery pain so that the mothers with a higher SES prevent from natural delivery. Our study showed that mother’s age and BMI were prognostic factors for CS. Similar results can be found in other studies (26, 27). Those with higher score of SES were more interested in CS. The CS rate differences were assessed among several categories of SES. Cesarean was extremely low among people in poor countries (28). The availability of medical and clinical utilities among high socio-economic population is one of the most important reasons for CS. In our study, baby’s head circumference was positively associated with cesarean. Large fetal head circumference is strongly associated with complicated labor and can increase the cesarean section rate. The association between postnatal head circumference and prolonged labor, signs of fetal distress and maternal distress was studied. The rate of each outcome increased gradually as the head circumference increased and emergency cesarean sections are expected after a large fetal head (29).

Three classification methods were performed and compared in this study. The chosen methods were based on generalized linear models, nonlinear dependency of the response variable to the predictors and non-parametric approaches. According to the highest accuracy as well as highest association of predicted and observed values for the CS, the ANN method outperformed in comparison to LR and RF. ANN methods perform non-linear statistical models and can be used to classify a case into a dichotomous response variable. Although complex computations are required for ANN, non-linear types of associations can be checked. This method is able to detect the interactions among several predictor variables (30). RF approach is appropriate when the number of predictor variables is high and it averages the results from several trees (19). LR is easy to use and interpret; complex associations provide difficulties in convergence and estimations, though. The outperformance of ANN in comparison to LR and RF can be due to the interactions among the predictors and a non-linear nature of association between CS and the predictors. However, several studies have compared the performance of different classification methods, these methods can differ in their efficacy of performances based on the data and the associations among variables. Classification methods have been compared in lots of clinical and medical data (31–33). The performance of logistic regression and artificial neural network was compared for estimating the risk of breast cancer and they found a similar performance of the methods and suggested using both of the models (34).Random forest, support vector machines, and artificial neural networks used to diagnose acute appendicitis. Random forest could predict acute appendicitis more accurate than other classification methods and can be an effective tool in clinical decision making (35).

## Conclusion

The rate of CS is considerably high in Iran which needs significant improvement in mothers’ education, psychological interventions to modify the attitude to CS, improving the quality of vaginal delivery services and eliminating the fear and anxiety of mothers about vaginal delivery. Moreover, ANN classification method was resulted as the best approach to classify a new case into CS based on its determinant factors such as baby’s head circumference, SES, BMI, infant weight and height.

## Ethical considerations

Ethical issues (Including plagiarism, informed consent, misconduct, data fabrication and/or falsification, double publication and/or submission, redundancy, etc.) have been completely observed by the authors.
